# Targeting endoplasmic reticulum associated degradation pathway combined with radiotherapy enhances the immunogenicity of esophageal cancer cells

**DOI:** 10.1080/15384047.2023.2166763

**Published:** 2023-03-12

**Authors:** Hui Luo, Yanan Sun, Liuxiang Wang, Hui Liu, Ran Zhao, Mengqiu Song, Hong Ge

**Affiliations:** a Laboratory of Radiation Oncology, the Affiliated Cancer Hospital of Zhengzhou University, Zhengzhou, China; b Academic of Medical Science, Zhengzhou University, Zhengzhou, China; c Department of Basic Medicine, China-US (Henan) Hormel Cancer Institute, Zhengzhou, China

**Keywords:** Esophageal cancer, immunogenic cell death, endoplasmic reticulum-associated protein degradation, inhibitor, radiotherapy, clinical outcomes

## Abstract

Immunogenic cell death (ICD) is essential for the activation of immune system against cancer. We aimed to investigate the efficacy of endoplasmic reticulum (ER)-associated protein degradation (ERAD) inhibitors (EerI and NMS-873) in enhancing radiation-induced ICD in esophageal cancer (EC). EC cells were administered with ERAD inhibitors, radiation therapy (RT), and the combination treatment. ICD hallmarks including calreticulin (CALR), adenosine triphosphate (ATP), and high mobility group protein B1 (HMGB1) were detected. The efficacy of ERAD inhibitors combined with RT in stimulating ICD was analyzed. Additionally, the role of ICD hallmarks in immune cell infiltration and patient survival was investigated. Inhibiting ERAD pathways was able to stimulate ICD component emission from dying EC cells in a dose-dependent pattern. Radiation-induced ICD was significantly increased after high doses RT (≥10 Gy). ERAD inhibitor combined with moderate dose RT (≥6 Gy) was capable of stimulating increased ICD in EC cells. Dual therapy could elicit the antitumor immune response by enhancing dendritic cells maturation and phagocytosis. Further investigation revealed a significant correlation between CALR and tumor-infiltrating immune cells. Low expression of ATP and HMGB1 and high expression of CALR were associated with favorable survival in patients with EC. The immunogenicityof EC can be enhanced by ERAD inhibitors combined with moderate doses of RT. ICD hallmark genes, especially CALR, are correlated to immune cell infiltration and clinical outcomes in EC. The present results demonstrated an important method to improve the immunogenicity of EC cells for enhanced antitumor immune response.

## Introduction

Esophageal cancer (EC) is a malignant tumor that occurs predominantly in the upper gastrointestinal tract. Current evidence indicates hot drinks, smoking, omission of vegetables and fruits, alcohol abuse, nitrosamines, gastroesophageal reflux disease, obesity, and human p virus infection are the primary risk factors of EC.^1^, ^2^ Antitumor treatments including surgery, chemotherapy, radiation therapy (RT), and immunotherapy were the main strategies for EC.^3^ Over the past few decades, RT and chemotherapy have played essential roles in locally advanced and advanced EC.^4^ Due to the lack of treatment response, tumor recurrence and distant metastasis were commonly observed in non-early-stage EC, and this eventually led to poor prognosis.^5^ Previous study described that the 5-year disease-free survival rate of locally advanced EC was less than 33%.^6^

Indeed, the existence of latent tumors could produce overt metastasis and local recurrence, resulting in treatment failure.^7^ The immune system is extremely important to eliminate latent tumors. RT could kill tumor cells by regulating anticancer immunity.^8^ Radiation-induced immune responses are often reliant on the immunogenicity of tumor cells.^9^ Enhanced efficacy of immunotherapy has been observed in dying tumor cells that underwent the liberation of immunogenic substances.^10^ However, several studies have reported the weak antigenicity of EC.^11,12^ Therefore, it is of great importance to enhance the immunogenicity of EC in the era of RT combined with immunotherapy.

Immunogenic cell death (ICD) is a key biological process in eliciting antitumor immune response.^13–15^ Briefly, ICD was characterized by the exposure of calreticulin (CALR) from endoplasmic reticulum (ER) to cytomembrane, the liberation of damage-associated molecular patterns (DAMPs) including adenosine triphosphate (ATP) and high mobility group protein b1 (HMGB1) into the tumor microenvironment.^14^ Tumor undergoing ICD was capable of inducing dendritic cells (DCs) maturation and triggering cytotoxic T cell-mediated adaptive immunity.^16^ Thus, ICD induction has emerged as a novel antitumor strategy. Notably, an essential role for cell stress has been revealed in all scenarios of ICD depicted thus far.^15^ The overloading of unfolded and misfolded proteins causes impaired ER homeostasis and results in ER stress.^17^ Then, the ERAD pathway was activated and involved in misfolded protein degradation to alleviate ER stress.^18^ Previous study demonstrated that RT alone was capable of inducing mild ER stress.^19^ Enhanced and prolonged ER stress was able to amplify ICD-associated immunogenicity in cancer; in contrast, weak or mild ER stress could be mitigated by the activation of ERAD pathway and had limited efficacy in triggering ICD.^18,20^ Thereby, targeting ERAD pathway may enhance radiation-induced ER stress and improve the immunogenicity of tumor cells. In recent years, several chemotherapeutic agents have been depicted to trigger ICD in various tumor models; however, the commonly used cytotoxic drugs in EC including fluorouracil and cisplatin were inefficient to induce ICD.^14^ Hence, it is vitally important to develop novel ICD triggering strategies.

In the present study, we characterized the role of ERAD inhibitors (EerI and NMS-873) in triggering ICD and evaluated the synergistic effects between ERAD inhibitor and moderate dose RT in stimulating ICD for EC cells. In addition, we attempted to clarify whether ICD hallmark gene expression is correlated with immune cell infiltration and patient survival in EC. These will provide a rationale for the application of combination strategies in inducing ICD, and thus enhance immunotherapy response in cancer patients.

## Results

### ERAD inhibitor reduces cell viability in EC cells

**Figure 1.**
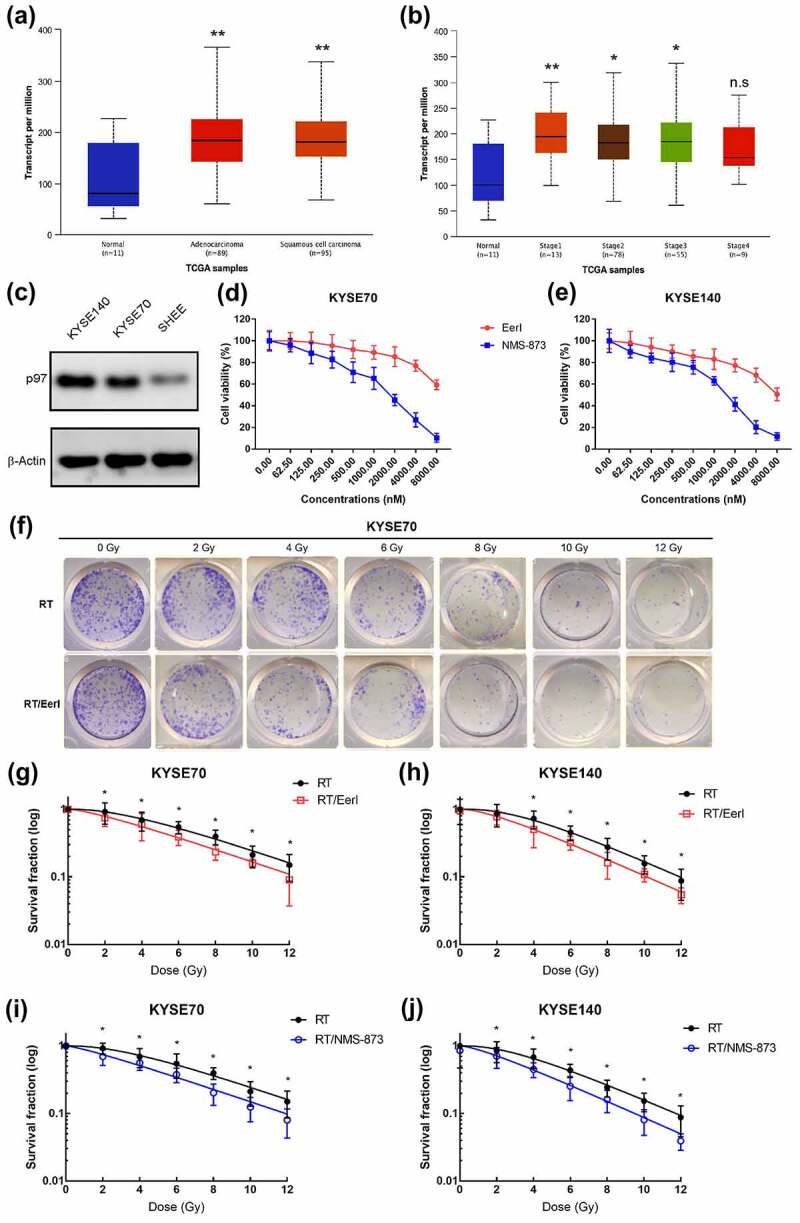
p97 was upregulated in EC, ERAD inhibitors decreases the growth of EC cells; targeting ERAD pathway enhances the cytotoxicity of RT in tumor cells. (a and b) TCGA database analysis of p97 levels in EC and normal tissues. (c) p97 expression in EC cells and normal human esophageal epithelial cells (SHEE). (d) Dose–survival curves of EerI or NMS-873 on KYSE70 cells were estimated by MTT assay at 24 hours. (e) Dose–survival curves of of EerI or NMS-873 on KYSE140 cells were estimated by MTT assay at 24 hours. (f) Representative images of the colony formation assays for KYSE70 cells treated with RT (0, 2, 4, 6, 8, 10, and 12 Gy), or RT/EerI (5000 nM). (g-h) Survival fraction curves of KYSE70 and KYSE140 cells following RT (0, 2, 4, 6, 8, 10, and 12 Gy), or RT/EerI (5000 nM) treatment. (i and j) Survival fraction curves of KYSE70 and KYSE140 cells following RT (0, 2, 4, 6, 8, 10, and 12 Gy) or RT/NMS-873 (500 nM) treatment. ERAD: endoplasmic reticulum associated protein degradation; EC: esophageal cancer; RT: radiation therapy; TCGA: The Cancer Genome Atlas. Data are means ± SD (*N* = 3), n.s, not significant, *P* > .05; *P ˂ 0.05; **P ˂ 0.01.

p97 has been depicted as a key regulator of the ERAD pathway. First, we estimated p97 expression in EC using TCGA database, and the results showed that p97 was significantly upregulated in EC tissues compared with normal tissues (Figure 1a). Despite there was no statistical significance in stage IV EC, p97 has been found to be highly expressed in early-stage and locally advanced EC (Figure 1b). We also detected p97 expression in ESCC cells and normal esophageal epithelial cells, and the results showed that p97 was highly expressed in both KYSE140 and KYSE70 cells compared with SHEE cells (Figure 1c). EerI is a potent ERAD inhibitor by suppressing the p97-Ufd1-Npl4 complex and impairing ataxin-3 dependent deubiquitination; NMS-873 is efficient in inhibiting p97 and suppressing the ERAD pathway.^21^ To ascertain the cytotoxic activity of ERAD inhibitors in EC cells (KYSE70 and KYSE140), we performed proliferation assay. The outcomes demonstrated that ERAD inhibitors including EerI and NMS-873 were capable of decreasing cell viability in a dose-dependent manner (Figure 1d and 1e). Moreover, NMS-873 was much more efficient than EerI. Using the cell viability assay, we found that 5000 nM EerI decreased the percent of cell viability from 100 %± 10.3% to 70% ± 5.1% in KYSE70 cells and 100% ± 9.6% to 62$ ± 4.8% in KYSE140 cells; besides, 500 nM NMS-873 reduced the percent of cell viability from 100% ± 7.7% to 68% ± 3.3% in KYSE70 cells and 100% ± 6.9% to 72% ± 2.9% in KYSE140 cells. These findings suggest that ERAD inhibitors have a deleterious effect on the proliferation of EC cells.

### Targeting ERAD pathway can synergize with RT and decrease cell colony formation

To evaluate the synergistic effect of ERAD inhibitor and RT in EC cells, we implemented colony formation assays (figure 1f). Dose survival curves indicated that pretreatment with ERAD inhibitor (EerI, 5000 nM; NMS-873, 500 nM) suppressed the clonogenic survival of KYSE70 and KYSE 140 cells exposed to different doses of irradiation. As shown in Figure 1g and 1h, when treated with EerI and different doses of RT, the survival fraction (SF) values were significantly decreased in KYSE70 cells. Although there was insignificant improvement for SF2 by the combination of EerI and 2 Gy of RT in KYSE140 cells, elevated doses of RT were associated with significantly enhanced SF. Meanwhile, in EC cells treated with NMS-873 and RT, the SF values were significantly reduced (Figure 1i and 1j). Taken together, these data clearly demonstrate that the combination of ERAD inhibitors and RT has an enhanced cytotoxic effect on EC cells.

### ERAD inhibitor and RT cause extracellular release of ATP from dying tumor cells

**Figure 2.**
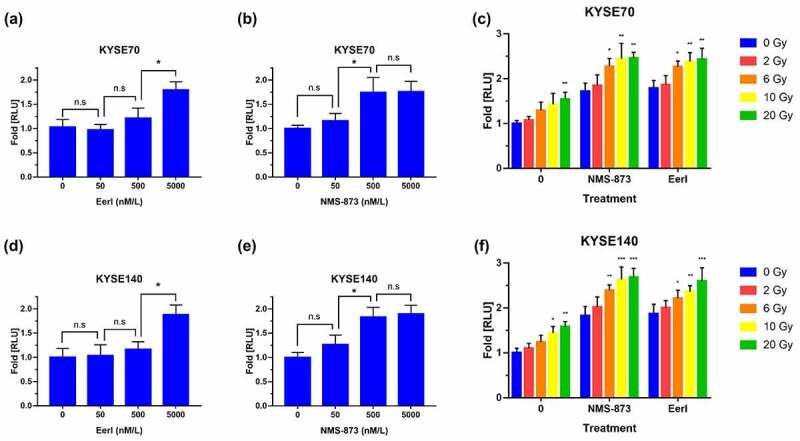
ERAD inhibitor and RT promote ATP release to extracellular space in EC cells. (a and b) Luminescence was measured after 24 hours exposure to increasing doses of EerI or NMS-873 in KYSE70 cells (0–5000 nM). (c) Luminescence was detected after 24 hours exposure to increasing doses of RT (0, 2, 6, 10, and 20 Gy) ± EerI (5000 nM) or NMS-873 (500 nM) in KYSE70 cells. (d-e) Luminescence was measured after 24 hours exposure to increasing doses of EerI or NMS-873 in KYSE140 cells (0–5000 nM). (f) Luminescence was detected after 24 hours exposure to increasing doses of RT (0, 2, 6, 10, and 20 Gy) ± EerI (5000 nM) or NMS-873 (500 nM) in KYSE140 cells. ATP: adenosine 5’-triphosphate; ERAD: endoplasmic reticulum associated protein degradation; EC: esophageal cancer; RT: radiation therapy. Data are means ± SD (*N* = 3), n.s, not significant,  *P* > .05; *P ˂ 0.05; **P ˂ 0.01; ***P ˂ 0.001.

ATP release into extracellular space is one of the hallmarks of ICD.^14^ Extracellular ATP could mediate DC maturation through the binding of P2X7 receptors, upregulating inflammasomes, and promoting inflammatory cytokines secretion.^22^ To determine the secretion of ATP among dying tumor cells in response to ERAD inhibitors, luminescent assays were conducted. As exhibited in Figure 2a and 2b, the RLU fold was increased in a dose-dependent manner after being exposed to different concentrations of ERAD inhibitors for KYSE70 cells. The amount of luminescence detected was significantly increased after being treated with 5000 nM EerI or 500 nM NMS-873 compared to the control group.

RT-induced ICD has been reported in several types of tumor cells.^14^ Similarly, RT promoted extracellular ATP release has been observed in the present study (Figure 2c). This effect appeared to occur in a dose-dependent fashion, and the emission of ATP increased rapidly with high-dose RT. Furthermore, we found that upon the addition of ERAD inhibitor, the RLU fold induced by RT remaining elevated. Although low-dose radiation-induced ATP was not significantly enhanced by ERAD inhibitors in KYSE70 cells, there was a significant synergistic effect between ERAD inhibitors and moderate-dose RT. In parallel, we evaluated the effect of RT and ERAD inhibitors on ATP release in KYSE140 cells, and similar outcomes have been attained (Figure 2d to 2f). Collectively, these data suggest that ERAD inhibitors and RT monotherapy triggered extracellular ATP release at the dosages tested in a dose-dependent manner. Targeting ERAD pathways combined with moderate doses of RT could synergize to enhance the liberation of ATP in EC cells.

### Treatment with ERAD inhibitor and RT induce CALR translocation to the surface of dying tumor cells

CALR, also known as ER resident protein 60, is a protein that binds to calcium ions; CALR acts as a molecular chaperone and involves in protein quality control and calcium ion homeostasis.^23^ Previous study reported that cancer cells undergoing RT were able to translocate intracellular CALR to their plasma membrane surface, served as an “eat me” signal, and resulted in ICD.^24^ However, such radiation-triggered CALR exposure on tumor cell surface was unsatisfied.

**Figure 3.**
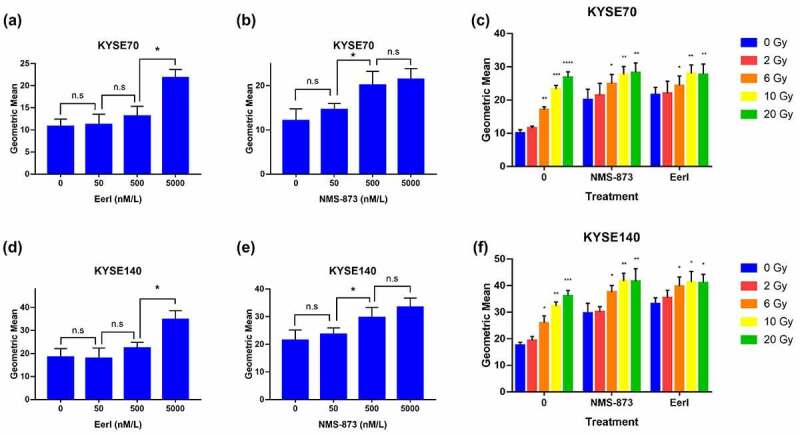
ERAD inhibitor and RT cause CALR exposure to the cell surface in EC cells. (a and b) Cell surface translocated CALR was measured after 24 hours exposure to increasing doses of EerI or NMS-873 in KYSE70 cells (0–5000 nM). (c) Cell surface translocated CALR was detected after 24 hours exposure to increasing doses of RT (0, 2, 6, 10, and 20 Gy) ± EerI (5000 nM) or NMS-873 (500 nM) in KYSE70 cells. (d and e) Cell surface translocated CALR was measured after 24 hours exposure to increasing doses of EerI or NMS-873 in KYSE140 cells (0–5000 nM). (f) Cell surface translocated CALR was detected after 24 hours exposure to increasing doses of RT (0, 2, 6, 10, and 20 Gy) ± EerI (5000 nM) or NMS-873 (500 nM) in KYSE140 cells. CALR: calreticulin; ERAD: endoplasmic reticulum associated protein degradation; EC: esophageal cancer; RT: radiation therapy. Data are means ± SD (*N* = 3), n.s, not significant, *P* > .05; *P ˂ 0.05; **P ˂ 0.01; ***P ˂ 0.001.

We next sought to confirm whether ERAD inhibitors could promote CALR cell surface exposure. EC cells were treated with ERAD inhibitors or RT, and cell surface CALR was calculated by flow cytometry (supplementary Figure 1A). The results demonstrated CALR translocation was dose-dependent. As displayed in Figure 3a to 3f, the amount of CALR exposed to cell surface was significantly elevated after being treated with 5000 nM EerI or 500 nM NMS-873 or high-dose RT compared to the control group. Interestingly, we noted an enhanced CALR translocation in EC cells treated with the combination of moderate-dose RT and ERAD inhibitor. In addition, membrane proteins were extracted, and the amount of cell surface CALR was detected by western blot. Our results confirmed the enhanced CLAR cell surface exposure after combination treatment (supplementary Figure 1B). Overall, these results indicate that suppressing ERAD pathways is capable of boosting radiation-induced CALR cell surface translocation in EC cells.

### ERAD inhibitor synergizes with RT and triggers HMGB1 secretion from dying tumor cells

After exposed to RT, HMGB1 was passively released from dying tumor cells into extracellular space and acted as a DAMP.^25^ Then, TLR4 on the surface of DCs binds to HMGB1 and initiates the antitumor immune response.^26^ Considering that ERAD inhibitors synergized with RT were able to trigger an enhanced tumor cell death, we wished to assess whether ERAD inhibitors could be utilized to promote HMGB1 release in EC cells.

**Figure 4.**
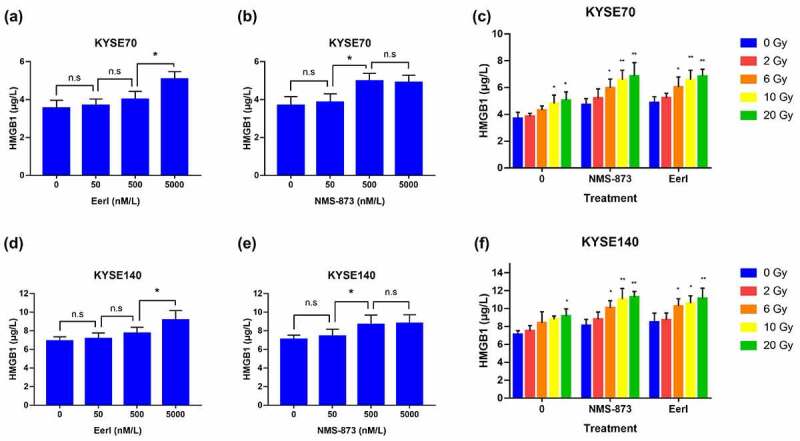
ERAD inhibitor and RT induce HMGB1 release to extracellular space in EC cells. (a andb) HMGB1 was measured after 24 hours exposure to increasing doses of EerI or NMS-873 in KYSE70 cells (0–5000 nM). (c) HMGB1 was detected after 24 hours exposure to increasing doses of RT (0, 2, 6, 10, and 20 Gy) ± EerI (5000 nM) or NMS-873 (500 nM) in KYSE70 cells. (d and e) HMGB1 was measured after 24 hours exposure to increasing doses of EerI or NMS-873 in KYSE140 cells (0–5000 nM). (f) HMGB1 was detected after 24 hours exposure to increasing doses of RT (0, 2, 6, 10, and 20 Gy) ± EerI (5000 nM) or NMS-873 (500 nM) in KYSE140 cells. ERAD: endoplasmic reticulum associated protein degradation; EC: esophageal cancer; HMGB1: high mobility group protein B1; RT: radiation therapy. Data are means ± SD (*N* = 3), n.s, not significant, *P* > .05; *P ˂ 0.05; **P ˂ 0.01; ***P ˂ 0.001.

As expected, the administration of ERAD inhibitors was correlated with enhanced HMGB1release on KYSE70 cells in a dose-dependent fashion (Figure 4a to 4b). RT alone was also associated with HMGB1 secretion, especially for high dose RT. Furthermore, the combination of ERAD inhibitors and moderate dose RT resulted in a remarkable HMGB1 release from dying tumor cells (Figure 4c). Concordantly, the results were confirmed on KYSE140 cells and clearly demonstrated increased levels of HMGB1 liberation after the delivery of RT and/or ERAD inhibitor (Figure 4d to 4f). As a whole, these results imply that both RT and ERAD inhibitors could significantly increase HMGB1 release in a dose-dependent manner among EC cells. The combination strategy has the potential to enhance HMGB1 secretion into the extracellular space.

### ERAD inhibitor combined with RT result in enhanced DCs phagocytosis and maturation

On the basis of the above data, the combination of moderate-dose RT and ERAD inhibitor was effective in eliciting ICD for EC cells. We sought to evaluate whether this combination strategy could trigger the adaptive arm of the immune system and facilitate cancer cell recognition and phagocytosing by DCs.

**Figure 5.**
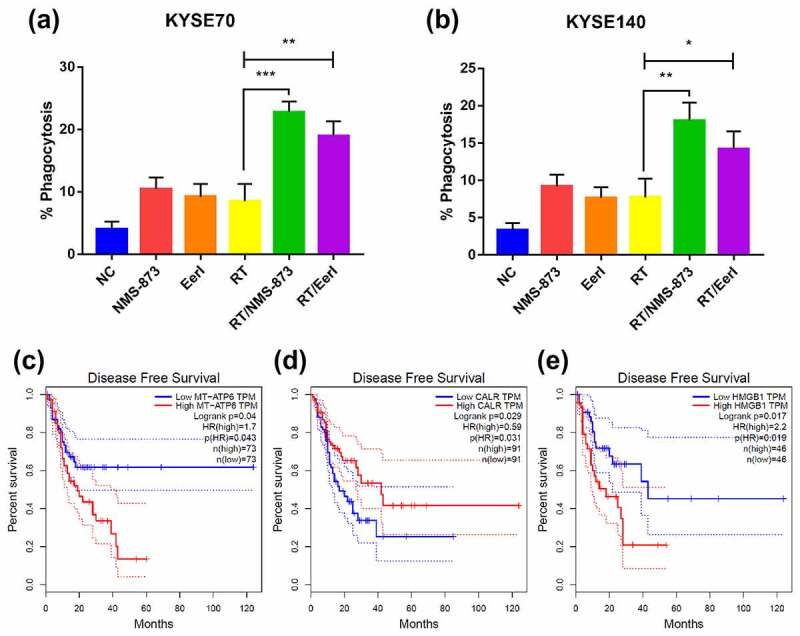
After treated with ERAD inhibitor and/or RT, phagocytosis assay showing the uptake efficacy of EC cells by DCs; the association between intracellular ICD hallmark gene expression and patient survival in EC were also analyzed. (a and b) Quantification was done for KYSE70 and KYSE140 cells engulfed by DCs after exposure to RT (6 Gy) ± EerI (5000 nM) or NMS-873 (500 nM). (c) Low MT-ATP6 expression (*N* = 73) is associated with favorable disease-free survival. (d) Low CALR expression (*N* = 91) is associated with poor disease-free survival. (e) Low HMGB1 expression (*N* = 46) is associated with favorable disease-free survival. CALR: calreticulin; DCs: dendritic cells; EC: esophageal cancer; ERAD: endoplasmic reticulum associated protein degradation; HMGB1: high mobility group protein B1; ICD: immunogenic cell death; MT-ATP6: Mitochondrially Encoded ATP Synthase Membrane Subunit 6; RT: radiation therapy. Data are means ± SD (*N* = 3), n.s, not significant, *P* > .05; *P ˂ 0.05; **P ˂ 0.01; ***P ˂ 0.001.

EC cells were efficiently engulfed by DCs within a few hours after treatment (supplementary Figure 1C). As illustrated in Figure 5a and 5b, compared with RT alone, a higher phagocytotic efficiency was observed in tumor cells treated with the combination regimen. Moreover, NMS-873 was much more efficient than EerI in assisting phagocytosis of EC cells by DCs.

Except for DC phagocytosis, the activation and maturation of DC was also important in eliciting an immune response. We analyzed the percentage of matured DCs by flow cytometry. Our results indicated that the expression of DC maturation markers including CD80 and CD86 was greater in the combination treatment group than in the monotherapy group (supplementary figure 1D to 1 G). Altogether, ICD-associated dying tumor cells could be efficiently phagocytosed by the DCs. ERAD inhibitors combined with RT are highly efficient in inducing DC maturation and enhancing ICD mediated antineoplastic immunity.

### The correlation between intracellular ICD hallmark gene expression and tumor-infiltrating immune cells

ICD was able to promote DC phagocytosis of tumor cells, induce macrophage polarization toward M1 type, stimulate the presentation of tumor-derived antigens, and resulted in CD8 + T cells activation.^16^ The CD4+ memory T cell could be produced during a primary immunogenic challenge.^27^ M1 macrophage represents antitumor activity whereas M2 macrophage promotes cancer progression and treatment resistance.^28^ With respect to immunogenic substances able to trigger the adaptive arms of immune system, we wished to assess whether there was a correlation between intracellular ICD hallmark genes and tumor-infiltrating immune cells in EC.

The TIMER2.0 server was applied, and as shown in supplementary figure 2, CALR expression was positively related to tumor-infiltrating immune cells including M1 macrophages (*R* = 0.160, *P* ˂ 0.05), DCs (*R* = 0.178, *P* ˂ 0.05), CD8+ (*R* = 0.164, *P* ˂ 0.05), and CD4+ (*R* = 0.243, *P* ˂ 0.01) memory T cells, whereas there was a negative correlation between CALR expression and M2 macrophages in tumor microenvironment (*R* = −0.15, *P* ˂ 0.05).

Besides, although a positive correlation between HMGB1 expression and CD4+ memory T cells (*R* = 0.195, *P* ˂ 0.01) was observed, the correlation coefficient of other tumor-infiltrating immune cells was insignificant. For genes involved in regulating the synthesis of ATP, the correlation coefficient for tumor-infiltrating immune cells was also not significant (data not shown).

Overall, the correlation between intracellular ICD hallmark genes and tumor-infiltrating immune cells implied an essential role of immunogenic substances, especially CALR, in triggering antitumor immunity.

### The association between intracellular ICD hallmark gene expression and patient survival

We next used the GEPIA database to explore the association between intracellular ICD hallmark gene expression and survival in patients with EC. The MT-ATP6 is an important subunit of ATP synthase, and contributes to the generation of cellular ATP.^29^ Based on the Kaplan–Meier survival analysis, patients with low expression of MT-ATP6 had a favorable disease-free survival compared to those with high expression of MT-ATP6 (HR [hazard ratio]: 0.264, *P* = 0.04) (Figure 5c). Conversely, patients with high expression of CALR had a longer disease free survival than those with low expression of CALR (*P* = 0.029) (HR: 0.59, Figure 5d). Nevertheless, patients with high expression of HMGB1 had a shorter disease free survival than those with low expression of HMGB1 (*P* = 0.017) (HR: 2.2, Figure 5e). These results indicated that intracellular ICD hallmark genes could be severed as prognostic factors in EC.

## Discussion

The development of ICD-inducing strategies are of great importance in the era of RT combined with immunotherapy. In the present report, we demonstrated that both ERAD inhibitor and RT were capable of eliciting the signals of ICD in a dose-dependent pattern. Although monotherapy with moderate doses of RT or ERAD inhibitors was insufficient to produce clearly observable immunogenic effects, targeting ERAD pathways enhanced radiation-induced ICD and facilitated tumor cell phagocytosis by DCs in EC cells. Further analysis suggested that ICD hallmark genes, especially CALR, were correlated with tumor-infiltrating immune cells and patient prognosis in EC.

In traditional radiation biology, radiation energy could be deposited directly into DNA molecules and resulted in cell damage or death, whereas indirect cell damage could be induced by the interaction between irradiation and water molecules.^30^ Nevertheless, radiation-induced bystander and abscopal effects have been reported in the last few decades.^31–34^ These novel radiation-mediated biological effects implied the potential evidence that RT was involved in the regulation of antitumor immune responses. Indeed, observable immunogenic effects have been depicted after hypofractionated RT.^35–37^ In the current report, we confirmed radiation-induced ICD in EC models, especially with high-dose RT.

**Figure 6.**
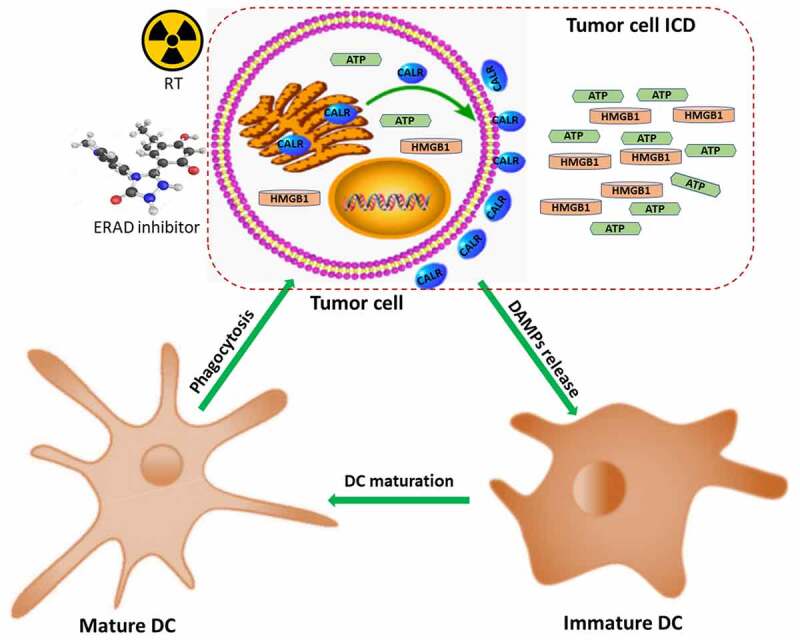
Mechanisms of ERAD inhibitor combined with RT in regulating ICD. ATP: adenosine 5’-triphosphate; CALR: calreticulin; DAMPs: damage-associated molecular patterns; DCs: dendritic cells; ERAD: endoplasmic reticulum associated protein degradation; ESCC: esophageal squamous cell carcinoma; HMGB1: high mobility group protein B1; ICD: immunogenic cell death; RT: radiation therapy.

ICD inducing agent concurrent with RT is one of the approaches to improve immunogenic effects. Anthracycline drugs have been widely used to manage and treat various types of malignant tumors.^38^ Doxorubicin, an anthracycline class medication, has been shown to promote the secretion of immunogenic substances from dying tumor cells.^39^ Unfortunately, there was limited effect of EC related chemotherapeutic drugs in improving ICD.^14,40^ The novelty of our analysis was that we demonstrate that suppressing ERAD pathway was associated with enhanced ICD in EC cells treated with RT. Mechanismally, ERAD inhibitors have a synergistic effect with RT and result in enhanced tumor cell death. Accordingly, the amount of each individual component of ICD was elevated from these dying tumor cells (Figure 6).

Dying cancer cells derived from ICD contributed to the activation of tumor microenvironment.^10,41^ It can be inferred that intracellular ICD hallmark gene expression may correlate with tumor-infiltrating immune cells. The current analysis revealed that CALR was positively related to M1 macrophages, DCs, CD8+, and CD4+ memory T cells in tumor microenvironment; conversely, CALR was negatively correlated with M2 macrophages. M2 macrophages represented an immune suppressive phenotype.^28^ Our results convincingly confirmed that CALR was crucial for stimulating antitumor immune response. Remarkably, there was insignificant correlation between intracellular HMGB1 expression and tumor-infiltrating immune cells; the correlation coefficient between genes involved in regulating the synthesis of ATP and tumor-infiltrating immune cells was also not significant. One possible explanation for the differing results may be that CALR has been reported to be overexpressed at the membrane level of cancer cells.^42^

Furthermore, the three cryptic immunogenic components have been evaluated as potential prognostic factors in various types of cancer.^14,42^ The present study confirmed that high expression of CALR and low expression of HMGB1 and MT-ATP6 were associated with favorable survival in EC. Notably, our prognostic analyses seemed inconsistent and there were several potential reasons for this. For weak immunogenicity tumors, HMGB1 and ATP were mainly expressed as intracellular pool, and only a small proportion was secreted extracellularly as immunogenic substances.^43,44^ Hence, these patients were associated with poor prognosis. In addition to the ER, a considerable amount of CALR was found on the surface of malignant tumor cells.^45^ Although CALR overexpression was associated with a higher density of immune cell infiltration and favorable survival in malignant cancer, whether these CALRs were expressed on cell surface or mainly from intracellular space remained unclear.^42^ Therefore, further investigation is needed to clarify the effect of intracellular CALR on cancer patient prognosis.

With the application of immune-checkpoint inhibitors, the efficacy of RT combined with immunotherapy has been confirmed in various malignant tumors.^46^ For tumors with high mutation burden and immunogenicities, such as renal cancer and melanoma, immunotherapy alone was enough to relieve immune suppressive effects on T cells; thus, patients could achieve survival benefits.^47^ In a phase II clinical study of preoperative chemoradiotherapy combined with pembrolizumab in the treatment of locally advanced EC, the pathologic complete response rate was 46.1% (NCT02844075).^48^ There was a positive correlation between pathological response rate and survival in EC patients. Insufficient radiation-mediated immunogenicity can also attenuate the antitumor effects of immunotherapy.^9^ Consequently, EC might be an excellent candidate disease for immunotherapy if immunogenicity can be further improved. Since a synergistic effect of ERAD inhibitor and RT in eliciting ICD was observed in the present analysis, the combinatorial therapeutic strategy may contribute to further enhancement of patients’ outcome for immunotherapy.

Although the present results revealed that ERAD inhibitors produced a dose-dependent induction and radiotherapeutic enhancement of ICD, potential limitations should be notified. The range of our *in vitro* assay is limited. The results demonstrated herein may be cell line-dependent. These factors may decrease the power of our analysis. Therefore, the present findings should be interpreted with caution, and additional studies including *in vivo* experiments are needed to support the results. Furthermore, previous studies indicated various cell death such as autophagy, apoptosis, and necrosis involved in mediating ICD.^49^ Rigorous studies are necessary to comprehend the molecular mechanism of ERAD inhibitors combined with RT in inducing ICD.

## Conclusion

In conclusion, our analysis suggests that both ERAD inhibitor and RT have a therapeutic dose-range that effectively stimulates the emission of immunogenic signals from dying EC cells. Targeting ERAD pathway could improve radiation-induced ICD, and this might facilitate anticancer effects in the immune system. There is a significant correlation between CALR and tumor-infiltrating immune cells. To date, immunotherapy focus primarily on immune checkpoint, low immunogenicity is one of the reasons for the limited therapeutic effects of existing immunotherapy drugs. Realizing the importance of immunogenic substances, researchers have been investigating the role of ICD in immunotherapy and, thus far, the results have been promising. Furthermore, the current study demonstrates that ICD hallmark gene expression could be used as a prognostic factor in EC. This will pave the way for the development of more effective ICD inducers. Much remains to be learned to delineate the mechanism of ERAD inhibitor combined with RT in triggering ICD and to translate these findings into clinics for more efficacious treatment results.

## Materials and methods

### Identification of key genes in ERAD pathway

The expressions of key regulators in ERAD pathway were retrieved from The Cancer Genome Atlas (TCGA) data portal. Genomic information of 184 EC tissues and 11 tumor-adjacent normal tissues was obtained. Key genes of ERAD pathway among normal tissues, tumor, and various tumor stages were analyzed.

### Cell lines and chemical reagents

Human EC cell lines (KYSE70, poorly differentiated invasive esophageal squamous cell carcinoma; KYSE140, moderately differentiated invasive esophageal squamous cell carcinoma) were purchased from the Type Culture Collection of the Chinese Academy of Sciences. SHEE cell line (an immortalized epithelium of the fetal esophageal epithelium) was obtained from Prof. Liu (China–US Hormel Cancer Institute). All the cells were maintained at 37°C with a humidified atmosphere of 5% CO_2_ and cultured in RPMI 1640 medium supplemented with 10% heat-inactivated fetal bovine serum (FBS) and 1% penicillin–streptomycin liquid. ERAD inhibitors including Eeyarestatin I (known as EerI, #3929, US) and NMS-873 (#6180, US) were purchased from Tocris Bioscience (UK).

### Cell proliferation assay

EC cells were seeded on 96-well plates with 3000 cells per well and incubated overnight at 37°C with a humidified atmosphere of 5% CO_2_. The next day, previous media were discarded, and wells were washed. The cells were administered with different concentration (0–8000 nM, 100 µl/well) of Eer I and NMS-873. After 24 hours, viable cells were incubated with 3-(4,5-dimethylthiazol-2-yl)-2,5-diphenyltetrazolium bromide (MTT 5 mg/ml, 20 µl/well) for 4 hours at 37°C. Then, 200 ul of DMSO was added to each well of the sample plate and incubated at 37°C for 2 hours. The absorbance value was quantified at 490 nm using a microplate reader (Thermo Scientific, US).

### Irradiation

EC cells were maintained in 10 cm plates and treated with various doses of irradiation (single fraction) using the 6 MeV photon beam (TrueBeam SN1403 accelerator, Varian Medical Systems, US). The following parameters were used: irradiation field, 13 cm × 13 cm; source skin distance, 100 cm; and dose rate, 3.9 Gy/min.

### Colony formation assay

Colony forming assay was conducted as previously described.^50^ Generally, tumor cells were seeded in 6-well plates with 200 cells per well. The ERAD inhibitors (EerI, 5000 nM; NMS-873, 500 nM) and/or various dose of RT (0, 2, 4, 6, 8, 10, and 12 Gy) were administered, then these cells were incubated for 2 weeks. The tumor colonies were fixated by 4% paraformaldehyde and stained with 1% glutaraldehyde-crystal violet solution and counted using the Image-proplus software (v.6.0) program (Media Cybernetics, Rockville, MD).^51^

### Luminescent ATP detection assay

Tumor cells (20000 cells per well) were seeded in six-well plates and maintained for 6 hours. Then, the cells were administered with ERAD inhibitors (EerI, 0–5000 nM; NMS-873, 0–5000 nM) or RT (0, 2, 6, 10, and 20 Gy) ± EerI (5000 nM) or NMS-873 (500 nM). After incubated for 6 hours, the supernatants were collected. Extracellular space ATP was measured by the Enhanced Luminescent ATP Detection Assay Kit (#S0027, Beyotime, China). First, the ATP working solution was prepared by using the ATP test solution and ATP diluent. After incubated for 3 minutes at room temperature, both samples (100 µL) and ATP working solution (100 µL) were added to the 1.5 ml EP tubes and mixed quickly. The amount of luminescence was quantified by the LuminoskanTM Ascent (465 nm, Thermo Scientific, USA) and reported as fold change in relative luminescent units (RLUs).

### Cell surface CALR expression by flow cytometry

The percentage of tumor cells expressing cell surface CALR could be measured by flow cytometry.^52^ Briefly, cancer cells (20000 cells per well) were seeded in six-well plates and incubated overnight. The next morning, the cells were administered with ERAD inhibitors (EerI, 0–5000 nM; NMS-873, 0–5000 nM) or RT (0, 2, 6, 10, and 20 Gy) ± EerI (5000 nM) or NMS-873 (500 nM), and maintained for 24 hours. The samples were harvested and resuspended with pre-cooled PBS at room temperature. After fixed for 10 minutes into the 3% formaldehyde and resuspended with incubation buffer, the cells were incubated with CALR rabbit monoclonal antibody (#12238, CST, USA) in 1.5 ml EP tubes (at room temperature for 1 hour). Cell centrifugation was performed, and the samples were incubated with Goat Anti-Rabbit IgG H&L (Alexa Fluor® 488) (ab150077, Abcam, UK) at room temperature for half an hour. Then, tumor cells were resuspended in pre-cooled PBS and analyzed using the 488 nm laser (50 mW, Argon-Ion Laser) of the flow cytometer (BD Biosciences, US).

### Extraction of membrane proteins

Briefly, EC cells were collected and washed three times with ice-cold PBS. Cell membrane proteins were isolated from the total membrane component by using the plasma membrane protein extraction kit (ab65400, Abcam, UK). Cell surface CLAR and sodium-potassium ATPase (Na+/K+ ATPase) were detected in the western blot.

### Western blotting

Western blot analyses were performed as described.^50^ Tumor cells were collected and lysed. The total protein concentration was determined by BCA assay following the manufacture’s instruction (Solarbio, China). The protein extracts were loaded onto the gel, separated by SDS-PAGE, and transferred to polyvinylidene difluoride membranes. After blocking with milk, the protein bands were incubated with diluted primary antibody (p97 Antibody #2648, calreticulin (D3E6) XP® Rabbit mAb #12238, GAPDH (14C10) Rabbit mAb #2118, β-Actin (13E5) Rabbit mAb #4970, Na,K-ATPase Antibody #3010, CST) and secondary antibody (Anti-rabbit IgG, HRP-linked Antibody #7074, CST). Finally, the membranes were imaged with an enhanced chemiluminescence detection reagent (Meilunbio, China).

### HMGB1 enzyme-linked immunosorbent assay (ELISA) assay

EC cells (20000 cells per well) were incubated in six-well formats overnight. Then, the cells were administered with ERAD inhibitors (EerI, 0–5000 nM; NMS-873, 0–5000 nM) or RT (0, 2, 6, 10, and 20 Gy) ± EerI (5000 nM) or NMS-873 (500 nM). After 24 hours of incubation, the supernatants were obtained, and Human HMGB-1 ELISA Kit (#D711210, Sangon Biotech, China) was used for the quantification of HMGB1. The results were expressed in terms of Optical Density (OD450) measurements using a microplate reader with an absorbance of 450 nm.

### Isolation of dendritic cells (DCs)

Freshly harvested blood samples were collected from healthy donors, and peripheral blood mononuclear cells (PBMCs) were isolated by means of Ficoll density gradient centrifugation. Macrophages were purified using the EasySep^TM^ Human CD11b positive selection Kit (STEMCELL Technologies Inc, Canada). Cells were then incubated in a medium supplemented with IL-4 (248 IU/ml, Gentaur, UK) and granulocyte macrophage colony-stimulating factor (500 IU/ml, Gentaur), and cultured for 5–6 days to obtain a population of immature DCs.

### Phagocytosis assay

EC cells were divided into untreated control, RT (6 Gy), ERAD inhibitors (EerI, 5000 nM; NMS-873, 500 nM), and the combination treatment group. After treated for 24 hours, the cancer cells were washed and labeled with CFSE (Abcam, UK). Next, tumor cells were cocultured with immature DCs at a ratio of 1:1 for 2 hours at 37°C. Then, mouse IgG1-phycoerythrin (PE) isotype control (1:1000, BD Biosciences, USA) or mouse anti-human CD11c-PE (1*10^6^ cells in a 100-µl experimental sample, BD Biosciences, US) were added to the cell mixture for 30 minutes at room temperature. After washing, the cells were assessed by a flow cytometer. The uptake of ICD tumor cells (phagocytotic efficiency) was calculated by the cell ratio of double positive cells (CFSE and CD11c-PE stained cells) in the CD11c-PE positive cells (DCs population).

### DC maturation and activation assay

Expressions of cell surface co-stimulatory molecules (CD80 and CD86) are markers of DC maturation.^53^ We assessed CD maturation and activation in EC cells. In brief, EC cells were treated with RT (6 Gy), ERAD inhibitors (EerI, 5000 nM; NMS-873, 500 nM), and the combination therapy, and maintained for 24 hours. Then, these cells were washed and cocultured with immature DCs at a ratio of 1:1 for at 37°C for 24 hours. To confirm the maturation of DCs, the harvested cells were stained with markers of maturation and activation (CD80-FITC, CD86-FITC, CD11c-PE; BD Biosciences, USA). The labeled cells were analyzed by flow cytometry.

### Analysis of tumor-infiltrating immune cells

The Tumor IMmune Estimation Resource (TIMER2.0) (http://timer.cistrome.org/) is an online program providing multiple types of cancer gene expression profiling for analyzing immune infiltration levels in TCGA cohorts.^54^ The correlation between ICD hallmark expression and tumor-infiltrating immune cells, including macrophages, DCs, CD8+, and CD4 + T cells, were analyzed by using the TIMER2.0 server.

### Survival analysis

The Gene Expression Profiling Interactive Analysis (GEPIA, http://gepia.cancer-pku.cn) is an online sever that provided comprehensive analysis of gene expression based on tumor and normal samples from Genotype-Tissue Expression (GTEx) and TCGA.^55^ The prognostic values of ICD hallmark were evaluated using the GEPIA. Disease-free survival was the primary endpoint and defined as the time from diagnosis to tumor recurrence or death. Kaplan–Meier plotter was applied for survival analysis.

### Statistical analysis

Each experiment was made in triplicate. The GraphPad Prism (version 7.0, GraphPad Software) was applied, and student’s *t*-tests, one-way ANOVA, or two-way ANOVA was used for statistical analysis. All the results were expressed as means (± standard deviation (SD)). Statistical significance was calculated as a two-tailed *P* ≤ 0.05 (n.s., non-significant; *P < 0.05; **P < 0.01; ***P < 0.001; ****P < 0.0001).

## Supplementary Material

Supplemental Material

## Data Availability

The authors confirm that the data supporting the findings of this study are available within the article.
