# Supplementary material for: Targeting endoplasmic reticulum associated degradation pathway combined with radiotherapy enhances the immunogenicity of esophageal cancer cells
Source: Cancer Biol Ther. 2023 Mar 12;24(1):2166763. doi: 10.1080/15384047.2023.2166763 (PMC10026871; doi:10.1080/15384047.2023.2166763)
Supplement: Supplemental Material [file KCBT_A_2166763_SM7903.zip › Figure_Legends_and_Alt_Text_for_Supplementary_Figures.docx]

**Supplementary Figure 1.** After treated with ERAD inhibitor and RT, cell surface exposed CALR was detected by flow cytometry and western blot, the uptake of EC cells by DCs was analyzed by flow cytometry. (A) Gating strategy diagram for flow cytometry analysis of CALR that translocated on the surface of EC cells. (B) The amount of CALR exposed to cell membrane were confirmed by western blot after treated with ERAD inhibitor (NMS-873, 5000 nM) and RT (6 Gy). (C) Flow cytometry study to investigate the uptake of EC cells by DCs after treated with ERAD inhibitor (NMS-873, 5000 nM) and RT (6 Gy). (D-E) Quantification of the expression levels of CD80 on DCs surfaces after exposure to RT (6 Gy) ± EerI (5000 nM) or NMS-873 (500 nM). (F-G) Quantification of the expression levels of CD86 on DCs surfaces after exposure to RT (6 Gy) ± EerI (5000 nM) or NMS-873 (500 nM). CALR: calreticulin; DC: dendritic cell; ERAD: endoplasmic reticulum-associated protein degradation; RT: radiation therapy.

**Supplementary Figure 1 Alt Text.** Different methods were applied to analysis CALR expression on the surface of EC cells after treated with ERAD inhibitors and/or RT. We also calculated dying EC cells that were engulfed by immune cells.

**Supplementary Figure 2.** The correlation between intercellular ICD hallmark gene expression and tumor-infiltrating immune cells were analyzed by using the TIMER2.0. (A-B) It revealed a positive correlation between the expression of CALR and tumor-infiltrating immune cells including M1 macrophages, DCs, CD8+ and CD4+ memory T cells, whereas there was a negative correlation between CALR expression and M2 macrophages in tumor microenvironment. (C-D) It revealed a positive correlation between the expression of HMGB1 and CD4+ memory T cells, whereas there was no correlation between HMGB1 expression and other tumor-infiltrating immune cells in tumor microenvironment. CALR: calreticulin; DC: dendritic cell; HMGB1: high mobility group protein B1; TIMER: Tumor IMmune Estimation Resource.

**Supplementary Figure 2 Alt Text.** Tumor-infiltrating immune cells have a relationship with ICD biomarkers.
