# Supplementary figures and images for: Targeting endoplasmic reticulum associated degradation pathway combined with radiotherapy enhances the immunogenicity of esophageal cancer cells
Source: Cancer Biol Ther. 2023 Mar 12;24(1):2166763. doi: 10.1080/15384047.2023.2166763 (PMC10026871; doi:10.1080/15384047.2023.2166763)

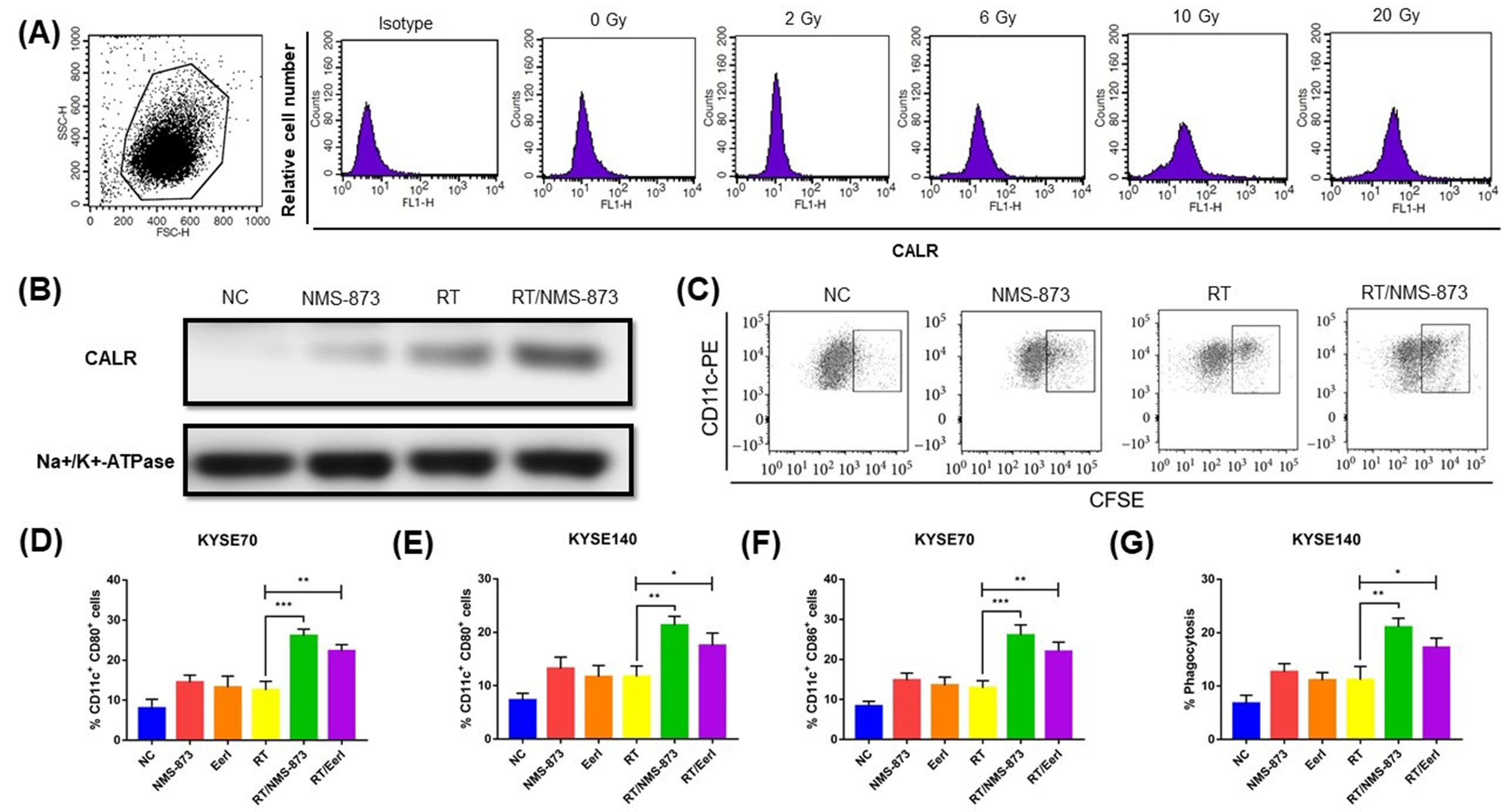

Supplement: Supplemental Material [file KCBT_A_2166763_SM7903.zip › Supplementary_Fig_1.tif]

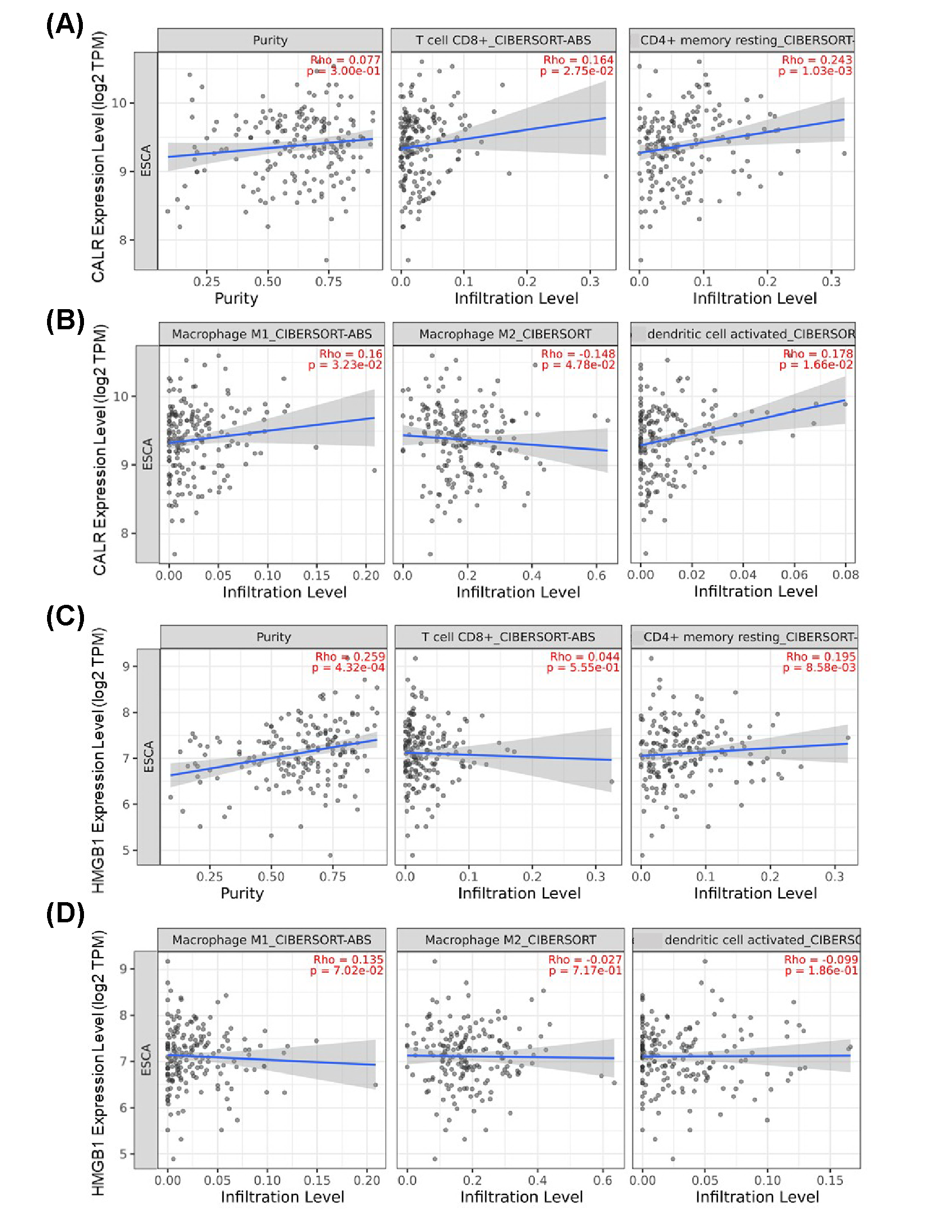

Supplement: Supplemental Material [file KCBT_A_2166763_SM7903.zip › Supplementary_Fig_2.tif]
